# Constipation of Berberis Vulgaris*Read before the Farrington Club of Pittsburg.

**Published:** 1889-05

**Authors:** John L. Ferson

**Affiliations:** Pittsburg


### CONSTIPATION OF BERBERIS VULGARIS.*

John L. Ferson, M. D. Pittsburg.

The constipation of *Berb.* is accompanied by hemorrhoids. The stools are pale in color, hard, and like sheep's dung; sometimes covered with blood. Much straining is required to expel them, due to a painful sensation of constriction in the rectum, which seems to prevent their escape. In consequence of this impediment there is a frequent urging to stool. The hemorrhoids are accompanied by itching or burning, particularly after stool. The anus is very sensitive to touch; the pressure on it when sitting causes pain, and there is pressure in the perineum. The absence of any statement as to whether the hemorrhoids protrude or are retained in the rectum, with the recording of the sensation of painful constriction in the rectum, and only soreness and sensitiveness of the anus lead me to conclude that the hemorrhoids of this drug are internal, and my comparisons will be made considering this as established. If any one knows differently let *us* know it. *Caust.*, *Chel.*, *Collin.*, *Mag-m.*, *Mez.*, *Op.*, *Plumb.*, and *Sepia.* all have stools similar in character to the one of Berb. under consideration.

The Caust. stool resembling that of Berb. is "light colored, hard, knotty, like sheep's dung." In color and condition very similar to Berb. Under *Caust.* the color may even be white. There is frequent urging to stool with both remedies. With

---

* Read before the Farrington Club of Pittsburg.

*Berb.* the urging is in great measure fruitless because of the sense of constriction in the rectum. With *Caust.* the urging is ineffectual because of a spasmodic, painful contraction of the sphincter ani; with the straining there is anxiety and redness of the face. Both remedies have hemorrhoids; but with *Caust.* they are external, protruding painfully. Both have itching and burning; *Caust.* having also sticking and stitching pains. *Berb.* has pressure in the perineum. *Caust.* has the same, and pulsations in the rectum.

CHELIDONIUM also has a stool like sheep's dung in size and form, and, like both *Berb.* and *Caust.*, the color is light. There is itching in the rectum with both; but there is a crawling sensation present with the itching under *Chel.* The hemorrhoids are absent. Jaundice is present under both remedies, and, although the urinary symptoms are not very similar, yet *Chel.* affects the kidneys quite markedly. The burning so prominently present in any part effected by *Berb.* is absent.

COLLINSONIA CAN. and *Berb.* both have constipation consisting of hard balls like sheep's dung, and of light color. Both are accompanied by internal hemorrhoids. Both have itching in the rectum, which is accompanied by heat in *Collin.*, while there is pronounced burning with *Berb.* *Collinsonia* has to distinguish it a sensation as of sticks or sand in the rectum, which makes it resemble Æsc. hip. With *Berb.* there is sometimes a covering of the stool with blood, while with *Collin.* there are frequently decided hemorrhages of dark, tough blood. There is no constriction in the rectum as with *Berb.;* instead is a sense of weight. The frequent urging of *Berb.* is absent.

MAGNESIA MUR. has a hard, knotty stool, like sheep's dung. Its color is not given. There is no hemorrhoidal condition present, as with *Berb.* There may be "much pressure to stool," as there is with Berb., but a more characteristic condition, in marked contrast is an atony of the bowel, without any desire, and this condition affects also the bladder, in which the urine accumulates without creating any urging to urinate, and its evacuation is only partly secured by great voluntary bearing down and pressure over the bladder. Both remedies have burning at the anus after stool, but there is no itching with *Mag-m.*

MEZEREUM causes a very hard, knotty, dark-brown stool in balls. In hardness resembling *Mag-m.* and *Plumb.* In a general way, aside from density and color, resembling Berb.

Hemorrhoids are not present, but there is an expulsion of the rectum with the straining which attends the efforts at defecation, weakness and laxity of the tissues being the cause both of the straining and the prolapse. The straining is painless, but the expulsion of the rectum may be followed by a constriction of the sphincter about it, which will cause swelling and intense pain. A fissure of the anus may be present to complicate matters, and when so, there is painful constriction of and drawing and tearing in the anus, extending to the perineum and through the urethra. There is much fetid flatus discharged, especially before stool.

The OPIUM stool is hard, dark, and of balls like sheep's dung; differing from *Berb.* only in color. Instead of the frequent urging and straining of *Berb.*, there is a paralyzed condition of the bowel which allows the accumulation of feces to go on for days without either urging or inconvenience. In some cases there is desire for stool, but when such is the case there is a sensation as if the anus was occluded; same as with Nux-v., but lacking the frequent urging of that drug (Jahr). There is marked dryness of the stool under Opium. The mucous membranes are likely to be dry from lessened secretion, this all through the intestinal canal, including the mouth. All the itching and burning, the constriction and sensitiveness, found under *Berb.* are absent.

PLUMBUM produces a very hard, dark stool, in ball like sheep's dung. Its resemblance to *Berb.* may even be closer, because under it the stool may lack bile, and be pale in color; and, to further heighten the similarity, jaundice may be present. Usually, however, the stools are dark with *Plumb.* The cause of the constipation is the same as with Opium—*i. e.*, paralysis of the muscular coats of the intestines, together with a dryness of the mucous membranes; but, unlike *Opium* and similar to *Berb.*, there is frequent urging to stool. This urging exists in spite of the fact that there is a paralyzed condition of the bowel, and is due to a constriction or spasm of the anus, even causing tenesmus.

A fissure of the anus may help along very materially in the way of pain. Note that the constriction is at the anus with *Plumb.*, while with *Berb.* it is in the rectum. With *Plumb.* there is a sensation as if the anus were being drawn up.

The abdominal symptoms of *Plumb.* are very characteristic of the drug, and, being so intimately associated with the symptoms of the stool, afford as easy a guide for the selection of the remedy in constipation as the urinary symptoms of *Berb.* do for that drug when they are present.

SEPIA has hard, knotty, scanty and insufficient stool, like sheep's dung. Unlike *Berb.* and indeed all the remedies considered, the stools are not dry, but are covered with mucus. The color is not mentioned.

As with *Berb.*, there is urgent desire to have a passage; attempts are unsatisfactory. Both remedies have straining in expelling the stool. With Sepia the weakness of the muscular coats of the rectum is such, that the expulsion of even a small quantity of mucous-covered feces requires great straining.

The scanty discharge from the bowels, in spite of the frequent urging and severe straining, gives slight if any relief; there remains so much of an accumulation that the urging continues, and there is present a sense of weight in the anus.

The straining causes, as with *Mez.* and *Plumb.*, a prolapse of the rectum, and the anus becomes swollen.

*Sepia* has burning of the anus like *Berb.*, but lacks the itching.

There is a constricting pain in the rectum and anus, which extends into the perineum or vagina (Dunham). This is similar to the constricting sensation in the rectum under *Berb.* Like Berb., Sepia has hemorrhoids, but they are external.

Distinctively *Sepia* has a "weak feeling in the abdomen after a stool; constant oozing of moisture from the rectum; and soreness between the buttocks."

